# Combined mirror visual and auditory feedback therapy for upper limb phantom pain: a case report

**DOI:** 10.1186/1752-1947-5-41

**Published:** 2011-01-27

**Authors:** Delia G Wilcher, Ivan Chernev, Kun Yan

**Affiliations:** 1 Boston University Medical Center, Department of Rehabilitation Medicine, 732 Harrison Avenue, F-511, Boston, MA, 02118-2398, USA; 2 Veterans Health Administration, Boston Healthcare System, Department of Physical Medicine and Rehabilitation, 1400 VFW Parkway AG 61, West Roxbury, MA, 02132, USA

## Abstract

**Introduction:**

Phantom limb sensation and phantom limb pain is a very common issue after amputations. In recent years there has been accumulating data implicating 'mirror visual feedback' or 'mirror therapy' as helpful in the treatment of phantom limb sensation and phantom limb pain.

**Case presentation:**

We present the case of a 24-year-old Caucasian man, a left upper limb amputee, treated with mirror visual feedback combined with auditory feedback with improved pain relief.

**Conclusion:**

This case may suggest that auditory feedback might enhance the effectiveness of mirror visual feedback and serve as a valuable addition to the complex multi-sensory processing of body perception in patients who are amputees.

## Introduction

There are over 130,000 limb amputations in the USA each year [1]. Nearly every amputee experiences some form of phantom limb effect, such as phantom sensation (voluntary or involuntary movements of the amputated limb, certain positions or sense of tactile stimulation of the amputated limb), telescoping, and/or phantom spasms. Additionally, a significant percentage of patients who are amputees may also experience phantom limb pain (PLP). The estimated prevalence of PLP varies from 49% to 83% [2]. PLP may negatively impact the quality of life of patients who are amputees and consume significant medical resources. The pathophysiology of phantom limb sensation and PLP is not yet well understood; however, complex peripheral and central mechanisms have been suggested [3]. Various types of treatments for PLP have been attempted, the outcomes of which have largely been disappointing.

Mirror therapy for phantom pain was first described by Ramachandran and Rogers-Ramachandran [4]. Mirror therapy has recently received more attention, with reports of an increased number of patients achieving beneficial outcomes [5-7].

The concept, also known as mirror visual feedback (MVF) has also demonstrated positive effects in other diseases such as stroke and complex regional pain syndrome [8,9]. As mirror therapy is based on visual feedback, it is possible that other types of stimuli such as auditory feedback may augment the treatment of PLP. To date, we know of no cases where combined mirror and auditory feedback therapy for PLP has been described. Here, we report a case of a left upper limb amputee treated with mirror therapy combined with auditory feedback.

## Case presentation

**Figure 1:**
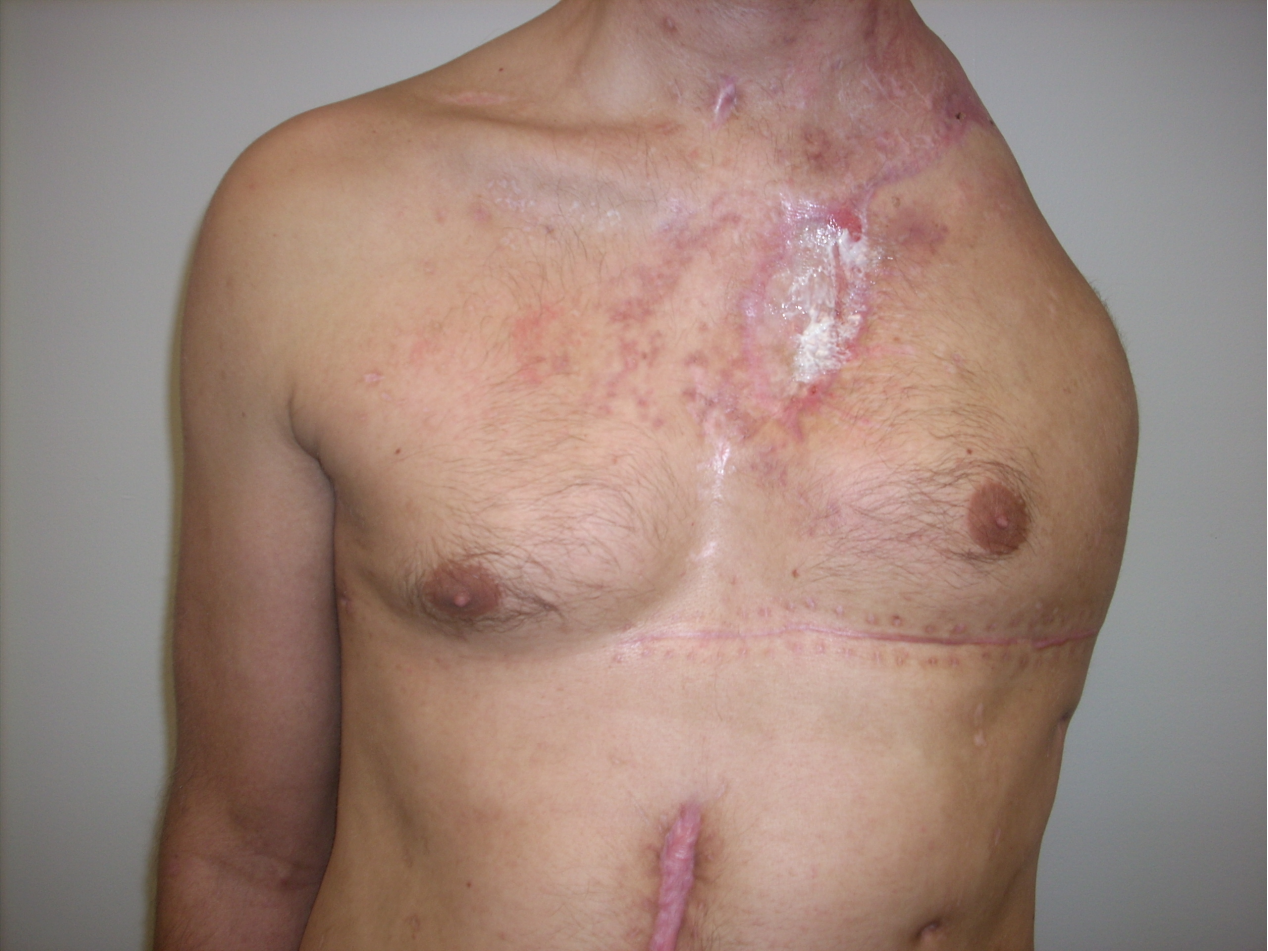
**Complete left upper limb amputation**. Digital photograph of post-traumatic anterior thorax demonstrating complete absence of left upper extremity and shoulder, 14 weeks after initial injury.

A 24-year-old Caucasian man, a full-time student, 1.8 m tall, 77 kg in weight, with no significant medical history, a non-smoker, taking no medications and with no substance misuse, was riding a motorcycle while wearing a helmet; he collided with a moving automobile and was ejected over 30 m into the air. He sustained multiple injuries including a large chest wall avulsion and a severe partial amputation of the left arm. The limb was not salvageable, requiring amputation, with a small residual fragment of the left scapula remaining (Figure 1). Left scapulothoracic dislocation and severed left brachial plexus were also found intra-operatively. His head, right arm and lower extremities were grossly intact.

He received 10 weeks of acute care in our surgical medical unit, where surgical intervention included repair of the chest wall and internal organs, after which he was transferred to the acute rehabilitation unit where, almost immediately, phantom limb pain became his major issue.

He reported his pain episodes as variable in number, ranging from three to six per day. Described as searing, aching or cramping as if his missing hand was clenched in a fist formation, the pain episodes often occurred at random intervals during the day, ranging from 15 minutes to up to an hour and a half. On average, he rated the pain at between 8 to 10 out of 10 on a visual analog scale (VAS).

As his entire left upper limb was missing, including the shoulder and parts of the clavicle and scapula, 'stump' pain did not actually apply to his description. Instead, he consistently experienced the feeling that his left fist was severely clenched and he could not release it from the cramping that became a burning, searing pain.

This persisted despite a series of aggressive pain management methods through the administration of naproxen 250 mg three times a day, tramadol 50 mg four times a day, extended release morphine 150 mg twice a day, hydrocodone/acetaminophen 5/500 mg every four hours as needed, lidocaine patches (two patches every 24 hours), gabapentin 400 mg four times a day and the use of a transcutaneous electrical nerve stimulation (TENS) unit. At this point our pain clinic was consulted for possible nerve block, which was deemed not appropriate. The pain was so severe that it affected patient's blood pressure as well. He required treatment with clonidine 0.4 mg twice daily, metoprolol 125 mg twice daily, and lisinopril 20 mg once daily. Over the course of two weeks, it was suggested that the employment of mirror therapy might provide some measure of relief. A vertically supported mirror in a frame was fashioned for easy positioning against his midline chest with him seated in a chair. In leaning slightly forward, he was able to watch the reflection of his right arm during motions as if doing biceps curls, opening and closing the fist, pronating and supinating the outstretched 'arms', while attempting to concentrate on doing these movements as if bilaterally. He performed these maneuvers for 15 minutes at a time at least twice daily. Although not significant in the first week to week and a half, he began to report some decrease in the intensity of the left upper extremity phantom limb pain by the end of the second week of the mirror therapy. He rated his maximal pain as 6 out of 10 on the VAS. All pain medications except gabapentin were gradually discontinued over two weeks of mirror therapy. Gabapentin was decreased to 400 mg three times a day.

His blood pressure also decreased after two days of mirror therapy. At the end of the third week he was only on lisinopril 20 mg daily.

During the mirror therapy course his mother participated by clapping her hands in synchrony with his movement of his hand towards the mirror, giving the illusion of not only seeing but also hearing hand clapping. We encouraged this form of auditory feedback and it was continued throughout his acute rehabilitation stay. Although MVF was started initially for the treatment of this patient's PLP, auditory feedback, at first performed unintentionally by his mother, was thereafter simultaneously performed along with the mirror therapy.

His other rehabilitation goals were met sooner than initially projected, and he was determined to be appropriate for discharge home with continuation of out-patient mirror and auditory feedback therapy, as well as further out-patient therapy care.

## Discussion

In the time since the phrase 'phantom limb' was introduced by Silas Weir Mitchell more than 130 years ago, hundreds of cases have been described. Many studies have sought to elucidate the pathophysiology in attempt to further develop treatments for phantom limb sensation and pain. The fact that it remains poorly understood, however, proves to be a hindrance.

In the non-amputee, signals sent from the motor and pre-motor cortex are verified by proprioceptive, sensory and visual feedback. In an amputee there is no verification, resulting in a conflict between the incoming and outgoing of information to the cortex. Interestingly, there is data showing that employment of a prosthesis has a therapeutic effect on PLP [9]. This could be due to the return of more sensory and proprioceptive feedback with the use of the prosthesis. In addition, mirror therapy may further enhance the sensory feedback through the illusory (mirror) image of the lost limb. Most of the published literature emphasizes the visual, sensory, and proprioceptive feedback with little or no mention of the auditory feedback created by familiar sounds such as hand clapping.

Recently discovered multi-sensory modulations, activations and connectivity at the earliest stages of perceptual processing may support a multi-sensory treatment approach to phantom limb and PLP, with the possibility of stimuli congruency contributing even further [10]. Shams and Seitz defined congruency as the relationship between stimuli that are consistent with the prior experience of the individual or relationships between senses found in nature [10]. For instance, the visual illusion of clapping hands is combined with an auditory feedback (the familiar sound of clapping hands) produced by a therapist or a third person. Although we did not use 'recorded' familiar sounds, it is likely that they could be employed as well. Another example could be snapping fingers, creating very specific sounds produced by our patient himself.

Although some sensory feedback might be more fundamental in limb perception than others, we believe that combined, congruent, multi-sensory stimuli are important in the overall process of perception of the phantom limb.

Whether the lessening of PLP in this case was due to the mirror therapy alone or to the combined MVF and auditory feedback is not clear. More cases utilizing multi-sensory feedback during treatment are needed to confirm this hypothesis.

## Conclusion

Multi-sensory feedback treatment may be superior to mirror therapy alone in the treatment of PLP in patients who are amputees. Further research is needed to explore the effects of multi-sensory stimulation in this patient population. We suggest that a controlled study comparing mirror therapy alone against combined MVF and auditory feedback may be beneficial in answering this question.

## Consent

Written informed consent was obtained from the patient for publication of this case report and any accompanying images. A copy of the written consent is available for review by the Editor-in-Chief of this journal.

## Competing interests

The authors declare that they have no competing interests. Portions of this case were previously presented as a poster at The Association of Academic Physiatrist Annual Meeting in Colorado Springs, February 2009, Colorado, USA.

## Authors' contributions

GDW performed data collection, participated in case writing, and critical review of the manuscript. IC participated in case writing, literature review, and critical review of the manuscript. KY participated in data collection, case writing and critical review of the manuscript. All have read and approved the final manuscript.
